# The economic burden of childhood pneumococcal diseases in The Gambia

**DOI:** 10.1186/s12962-016-0053-4

**Published:** 2016-02-17

**Authors:** Effua Usuf, Grant Mackenzie, Sana Sambou, Deborah Atherly, Chutima Suraratdecha

**Affiliations:** MRC, The Gambia Unit, PO Box 273, Banjul, Gambia; Pneumococcal Group, Murdoch Children’s Research Institute, Parkville, Australia; Faculty of Infectious and Tropical Diseases, London School of Hygiene and Tropical Medicine, London, UK; Ministry of Health, Banjul, Gambia; PATH, Seattle, USA; U.S. Centers for Disease Control and Prevention, Atlanta, USA

## Abstract

**Background:**

*Streptococcus pneumoniae* is a common cause of child death. However, the economic burden of pneumococcal disease in low-income countries is poorly described. We aimed to estimate from a societal perspective, the costs incurred by health providers and families of children with pneumococcal diseases.

**Methods:**

We recruited children less than 5 years of age with outpatient pneumonia, inpatient pneumonia, pneumococcal sepsis and bacterial meningitis at facilities in rural and urban Gambia. We collected provider costs, out of pocket costs and productivity loss for the families of children. For each disease diagnostic category, costs were collected before, during, and for 1 week after discharge from hospital or outpatient visit.

**Results:**

A total of 340 children were enrolled; 100 outpatient pneumonia, 175 inpatient pneumonia 36 pneumococcal sepsis, and 29 bacterial meningitis cases. The mean provider costs per patient for treating outpatient pneumonia, inpatient pneumonia, pneumococcal sepsis and meningitis were US$8, US$64, US$87 and US$124 respectively and the mean out of pocket costs per patient were US$6, US$31, US$44 and US$34 respectively. The economic burden of outpatient pneumonia, inpatient pneumonia, pneumococcal sepsis and meningitis increased to US$15, US$109, US$144 and US$170 respectively when family members’ time loss from work was taken into account.

**Conclusion:**

The economic burden of pneumococcal disease in The Gambia is substantial, costs to families was approximately one-third to a half of the provider costs, and accounted for up to 30 % of total societal costs. The introduction of pneumococcal conjugate vaccine has the potential to significantly reduce this economic burden in this society.

**Electronic supplementary material:**

The online version of this article (doi:10.1186/s12962-016-0053-4) contains supplementary material, which is available to authorized users.

## Background

There was an estimated 826,000 deaths due to pneumococcal diseases. In the year 2000, representing more than 11 % of all childhood deaths with 90 % of deaths from pneumococcal pneumonia occurring in Africa [1]. In 2013 pneumonia remains a leading cause of death contributing 15 % of the total global child deaths [2]. The pneumococcus is the most common cause of pneumonia, sepsis, and meningitis [3]. In addition to high case fatality rates, survivors of pneumococcal meningitis often experience disability such as cognitive impairment, seizures and deafness [4, 5].

In spite of the extensive disease burden, data describing the economic burden of pneumococcal disease in sub-Saharan Africa are limited. A study in Kenya estimated the treatment cost for episodes of pneumonia in children to vary from US$54 to US$99 in regional and district hospitals and up to US$177 in the national hospital. For meningitis, the estimated costs were higher, US$189 to US$201 and up to US$284 in the national hospital [6]. Another study in Zambia found the treatment costs of outpatient and inpatient pneumonia to be US$48 and US$215 respectively [7]. During a clinical trial of a pneumococcal conjugate vaccine (PCV) in The Gambia, the average treatment cost of pneumococcal illness was estimated at US$191 [8].

Most studies of the economic burden of disease or vaccine cost-effectiveness do not collect primary data on costs but instead rely upon other sources such as estimates from neighbouring countries often with a number assumptions [9, 10] or the ‘WHO-CHOICE’ (choosing interventions that are cost-effective) database of cost estimates for hospital care [11]. However, several studies have demonstrated that the WHO-CHOICE cost estimates generally underestimate the true cost when compared to micro costing methods [11, 12].

Effective PCVs are now available and with the help of Gavi, the Vaccine Alliance, these vaccines are becoming part of routine immunisation in many developing countries [13]. In 2009, The Gambia was one of the first countries in Africa to introduce PCV. Pneumococcal vaccination is associated with high investment costs. An assessment of the likely economic benefits of PCV is essential in settings with limited resources [14]. Determining the cost-effectiveness of different vaccines will assume greater importance as immunisation programmes add more expensive vaccines such as PCV and rotavirus vaccine, to the less expensive, traditional vaccines such as measles, oral polio and BCG.

A study to determine the impact of PCV in The Gambia has been underway since 2008 [15]. Our economic evaluation has already estimated the cost of introducing PCV into the national Expanded Programme of Immunisation (EPI) [14]. In order to provide accurate estimates of the economic burden of pneumococcal disease we used micro-costing methods with empiric, local, data, including costs to both the health care provider and families. We also conducted sensitivity analyses and we provide a national estimate of the economic burden of disease. These findings provide critical inputs to evaluate the overall cost-effectiveness of introducing PCV.

## Methods

### Setting

The study was undertaken in 2011 and 2012 at two sites, Basse Health Centre (Basse), in a rural town in upper river region (URR), The Gambia and Royal Victoria Teaching Hospital (RVTH) recently renamed Edward Francis Small Teaching Hospital in Banjul, the capital city. The health centre at Basse serves as a referral centre for all minor health centres in URR and RVTH serves as the main tertiary hospital for all regions.

Treatment of children aged under 14 years in The Gambia is provided free of charge. Case management of pneumonia follows WHO’s Integrated Management of Childhood Illness strategy. Even though the costs directly related to treatment at health facilities are covered by the government, families incur out-of-pocket expenses and loss of income before presentation to hospital, while taking care of a sick child at hospital and after discharge. Additionally, sequelae following pneumococcal meningitis are common and result in substantial economic burden to families.

### Participants

Children were potentially eligible for the study if they were aged less than 5 years and attended Basse or RVTH. Patients attending RVTH were assessed for eligibility by study staff on a daily basis between 9am and 2pm. Children were given treatment at the outpatient department (OPD) or admitted to the ward according to the discretion of the attending clinician. Patients in Basse were enrolled in a surveillance project to monitor individuals with pneumonia, sepsis and meningitis [15]. Surveillance staff used standardised criteria to screen and refer patients with possible pneumonia, sepsis, or meningitis to surveillance clinicians who then assessed and investigated children according to standard criteria and admitted patients according to their clinical judgement. We enrolled the caregivers of children in four diagnostic categories: (1) a clinician’s diagnosis of clinical pneumonia treated as an outpatient, (2) a clinician’s diagnosis of clinical pneumonia admitted to the ward, (3) an illness in which *Streptococcus pneumoniae* was isolated from blood, and (4) an illness in which pathogenic bacteria was isolated from cerebrospinal fluid.

### Data collection

After confirmation of eligibility and informed consent, study staff interviewed the child’s caregiver. For hospitalised patients, data were collected for the day of admission and any days of illness prior to this first interview. Subsequently, caregivers were interviewed on a daily basis to obtain retrospective data during the previous 24 h, until the day of discharge. Caregivers of children treated at the OPD were interviewed on the day of presentation and after the discharge of patients. Staff conducted a home visit 7 days after discharge or outpatient visit and collected data on costs incurred during the days following discharge/visit until the day of the home visit. All data were recorded on standardised questionnaires.

We collected data in order to determine costs per case. Data included staff cost, clinical consumables, drugs consumed by each patient, diagnostic tests, the number of days on intravenous fluid, number of days on oxygen therapy, and the length of hospital stay. We used the unit costs of each medication provided by the national pharmaceutical service which procures and distributes drugs to all government facilities (Additional file 1: Table S1). We used either the dose-specific cost or the full cost, depending on whether a drug vial could be used for additional patients or had to be discarded once it was opened and partially used. For blood transfusion we used the reported cost of providing one unit of blood from the national blood transfusion service, US$9.02 (personal communication, Jaye P. 2011). We estimated the cost of oxygen by using the purchase price of an oxygen concentrator, its expected shelf life and the ratio of oxygen cost when provided by concentrator compared to oxygen cylinders as determine by Howie et al. [16]. The average cost of 1 day of oxygen therapy was estimated to be US$1.95. The costs of diagnostic tests were derived from the Medical Research Council, The Gambia Unit (MRC). Since the RVTH user charges for older children and adults are heavily subsidised by the government, we used the MRC estimates which take into account all relevant cost components. We collected costs from private laboratories and compared these with the MRC laboratory costs (Additional file 1: TableS2) for inputs in a scenario analysis.

#### Costs per hospital bed day

The resources used at both study sites are summarised in Additional file 1: Table S3. Bed occupancy, the average proportion of beds that are occupied at any one time, was calculated as the total number of admissions multiplied by the average duration of admission in days, divided by 365. Dividing this average number of patients per day by the number of beds gives the bed occupancy rate. We used the government salary scale and lists of facility personnel and their grades to calculate the daily cost of personnel in each facility. We excluded the cost of buildings, maintenance, cleaning and utilities as these data were not readily available. Market costs of locally purchased foodstuffs and the weekly menu of meals were used to estimate the daily cost of food provided by the hospital and local prices and the daily volume of materials consumed were used to estimate the daily cost of laundry.

#### Family out-of-pocket costs

Out-of-pocket costs to caregivers and other family members were estimated for three periods, before and during hospitalisation, and for 1 week after discharge. Data collected included the following: costs of diagnostic tests, consultations, drugs and transportation before presentation to the facility, food consumed during the hospital stay, its source and the actual cost or an estimate if it was not purchased, the source of money which was used by the caregiver to pay for the costs and total household expenditure. We also recorded the costs of burial at RVTH. Additionally, we estimated time loss by caregivers and visitors due to travelling between home and facility, and while the patient received treatment at the health facility,

#### Costs associated with bacterial meningitis sequelae

Children aged 5 years or less with confirmed bacterial meningitis were recruited separately to estimate the costs associated with sequelae following pneumococcal meningitis. In the coastal area, these children were recruited retrospectively from patients who were enrolled in an MRC meningitis surveillance project between 2008 and 2010. These children were enrolled within 24 months after the illness. Staff enquired about the child at the given address and sought informed consent from the caregiver if the child was identified. Children in Basse with meningitis were enrolled prospectively as part of the surveillance study but caregivers were only approached for economic burden of meningitis sequelae data 6 months after the acute illness.

For both retrospective and prospective cases, we only recorded data up to 6 months post discharge. We recorded the duration of the admission and the estimated amount of money spent before, during and for 1 week after discharge from hospital. We also recorded the degree to which the child was able, or unable, to perform normal activities and treatment costs during the period from 1 week and up to 6 months after discharge. The level of care provided and the relationship with the caregiver was recorded. Payments for someone to take care of the child or loss of income for those providing care were estimated based on their likely income if they were employed.

### Health facility surveys

To assess the generalisability of data collected at RVTH and Basse to other health facilities in the country, we interviewed the nurse in charge at RVTH, Basse, one other hospital from the remaining five regional hospitals and at four out of approximately 50 other public health centres spread across the country. Selection of health facilities used a purposeful approach according to number of government facilities of different sizes in rural and urban regions. We collected data on standard treatment protocols, available diagnostic tests, staffing levels, provision of food, and numbers of patients admitted in 2010 with pneumonia, sepsis and meningitis.

### Ethics

All caregivers who participated provided written informed consent. Caregivers read the project information sheet or staff read the sheet in a language that the carer understood. After consent the caregiver signed a form, or provided their thumb print. Nurses at the surveyed health facilities gave verbal consent after reading the information sheet provided. The Gambia Government/MRC Joint Institutional Ethics Committee approved the study (SCC 1211), as did the PATH Research Ethics Committee (HS 560).

### Analysis

We aimed to collect cost data from a sample of 100 pneumonia cases at each centre, 50 inpatient and 50 outpatient. Assuming a mean cost of U$100 per case with a standard deviation of US$33 [6], enrolment of 50 patients in each category would provide an estimate with precision of ± US$6.5. Given the limited number of sepsis and meningitis cases, the sample size was based on the number of cases identified.

All costs were presented in total, mean, median, and inter quartile range (IQR) by facility and by diagnosis. To determine the total provider costs, the estimated average treatment cost for each diagnostic category was added to the estimated bed day cost multiplied by the average duration of admission for each diagnostic category. Estimation of costs to the caregiver and family associated with sequelae following bacterial meningitis was calculated in a similar manner. We placed a value on the time loss based on the daily wage or how much the caregiver would pay for the work to be carried out, whichever was provided in the questionnaire, both options were available [17]. The total societal cost was a summation of the costs to the provider, costs borne by caregivers and families for each diagnostic category and the productivity loss of family members. All costs were estimated in US$ dollars using the average exchange rate for the year 2010.

### Sensitivity analysis

Two different scenarios were analysed. One scenario assumed lower levels of access to diagnostic tests. Given the use of X-ray and microbiology investigations in Basse was greater than at the RVTH and other facilities, a sensitivity analysis was conducted based on utilization of these investigations at RVTH. In a second scenario, we compared costs for diagnostic test from private laboratories to costs at the MRC which were used in the base case analysis.

## Results

### Characteristics of patients

A total of 340 patients were recruited, 197 (57.9 %) from Basse and 143 (42.1 %) from RVTH. There were more males at both sites. Overall 57.4 % of the children were male. The median age (IQR) in months was 9 (4–18) and 10 (4–24) in Basse and RVTH respectively. The age distribution was not different between the two sites (p value = 0.41). Table 1 shows the number of patients recruited in each diagnostic category, from each facility.

**Table 1:** Number of children recruited and the number that died in each diagnostic category

Diagnosis	Basse N (%)	RVTH N (%)	Total N (%)	Deaths N (%)
Outpatient pneumonia	50 (25.4)	50 (35.0)	100 (29.4)	0 (0.0)
Inpatient pneumonia	94 (47.7)	81 (56.6)	175 (51.5)	10 (5.7)
Pneumococcal sepsis	32 (16.2)	4 (2.8)	36 (10.6)	10 (27.8)
Bacterial meningitis	21 (10.7)	8 (5.6)	29 (8.5)	16 (55.2)
Total	197 (100.0)	143 (100.0)	340 (100)	36 (10.6)

The average inpatient stay was 5.8 days; 4.6 days in Basse and 7.6 days at RVTH. The maximum stay was 35 days. Thirty-six (10.6 %) children died and one absconded from hospital. Ten (4.2 %) children died on the same day they were admitted. The children who died had an average length of stay of 3.9 days compared to 6.1 days for children who survived (p value = 0.02). Deaths were more common among the meningitis cases 16/29 (55.2 %) compared to sepsis (10/36) and pneumonia (10/175) cases (p value < 0.001). No patient who was treated for pneumonia as an outpatient died during the study period.

### Characteristics of caregivers and households

The mother was the caregiver for 90.6 % of cases and the father for 6.8 %. On average, there were 12 (IQR 6–15) adults and 15 (IQR 7–20) children aged under 18 years per household in Basse and 5 (IQR 3–7) adults and 6 (IQR 3–7) children per household in urban areas. The average daily household expenses were greater in Basse at US$9.05, (median, IQR US$7.35, 3.7–11.0) than RVTH at US$5.40, (median, IQR US$3.70, 2.7–5.9). The per capita expense per day based on the size of the household however was US$ 0.49 and US$ 0.33 at Basse and RVTH respectively.

Thirteen percent of households (8 % in rural area and 18.8 % in urban area) reported that there were one or more days in the previous month when there was no food in the house. The source of income to cover medical expenses was exclusively from current income in 25.7 % of households, savings 21.0 %, cutting down other expenses 10.1 %, and borrowing or donations from relatives and friends 6.2 and 18.5 % respectively. Selling assets was not common (1.1 %) and 17.4 % had a combination of financing strategies.

### Provider costs

The paediatric bed occupancy rate at RVTH and Basse were 0.88 and 0.95 respectively. Aggregating the estimated costs per patient day for food, laundry, and personnel, the estimated cost per paediatric patient day at Basse was US$5.29 and US$4.37 in RVTH.

The distributions of costs were generally right-skewed. The average total provider treatment cost for outpatient pneumonia, inpatient pneumonia, pneumococcal sepsis and bacterial meningitis were US$5.50, US$60.90, US$90.90 and US$131.00 in Basse and US$9.80, US$67.90, US$51.60 and US$104.20 at RVTH respectively per patient (Table 2). Fifty-three children (15.6 %) received three or more antibiotics during the treatment.

**Table 2:** Provider hospital costs; patient specific and non-specific costs (bed day costs) US$

	Mean LOS days^a^	Costs mean, median (IQR)					
		Patient specific costs				Bed day costs^e^	Total costs^f^
		All drugs^b^	Investigations	Others^c^	Subtotal^d^		
Basse							
Outpatient pneumonia n = 50	n/a	2.5, 1.3 (0.8–2.0)	3.0, 3.1 (1.9–5.0)	n/a	5.5, 4.6 (3.2–5.9)	n/a	5.5, 4.6 (3.2–5.9)
Inpatient pneumonia n = 94	4.3	9.0, 5.2 (3.2–10.3)	28.2, 23.2 (21.1–26.5)	1.3, 0 (0–1.8)	39.0, 31.1 (26.5–41.5)	21.8, 15.9 (15.9–26.4)	60.9, 49.7 (43.0–72.3)
Sepsis n = 32	5.7	11.3, 5.5 (2.5–10.1)	48.8, 26.6 (23.2–73.0)	0.9, 0 (0–0)	61.0, 54.0 (27.9–82.5)	24.9, 26.4 (15.9–27.3)	90.9, 86.0 (54.7–121.5)
Bacterial meningitis n = 21	5.1	15.4, 6.6 (1.2–23.2)	85.2, 105.4 (69.9–118.0)	3.4, 0.6 (0–2.34.0)	104.0, 113.2 (71.1–131.5)	27.0, 26.4 (5.3–43.3)	131.0, 123.7 (112.3–169.2)
RVTH							
Outpatient pneumonia n = 50	n/a	3.2, 1.0 (0.5–2.4)	6.5, 5.4 (5.4–8.5)	n/a	9.8, 7.8 (6.1–12.8)	n/a	9.8, 7.8 (6.1–12.8)
Inpatient pneumonia n = 81	7.8	16.8, 3.2 (1.8–7.8)	13.3, 8.5 (5.4–8.5)	3.6, 1.8 (0–5.4)	33.7, 15.3 (10.3–29.4)	34.2, 26.2 (17.5–35.0)	68.0, 43.5 (30.7–71.8)
Pneumococcal sepsis n = 4	5.0	9.1, 2.9 (1.1–17.0)	11.5, 6.9 (5.4–17.6)	9.2, 2.0 (0.8–17.6)	29.8, 34.8 (18.4–41.2)	21.9, 10.9 (9.4–39.3)	51.6, 39.2 (29.3–74.0)
Bacterial meningitis n = 8	6.6	43.3, 6.7 (3.2–37.7)	28.4, 11.9 (5.4–56.2)	3.5, 2.9 (0.8–6.5)	75.2, 54.8 (16.2–98.6)	29.0, 21.8 (13.1–37.1)	104.2, 85.4 (35.9–120.5)

*Los* length of stay in hospital, *IQR* interquartile range

^a^ Ten died on the same day they were admitted, length of stay for these estimated as 1 day to cover nonspecific patient costs

^b^All drugs antibiotics and other non-essential drugs

^c^Others- intravenous fluids (IVF), blood transfusion, oxygen

^d^Subtotal all patient specific costs

^e^Non-specific costs such as cleaning, personnel

^f^Total costs patient specific cost plus bed day costs

### Family out-of-pocket costs

Table 3 summarises the mean out-of-pocket costs by disease category. Half (174/340) of caregivers had consultations elsewhere before presenting to RVTH or Basse. The mean cost to families before hospitalisation in Basse was US$1.60 (95 % CI 1.1–2.1) and US$7.50 (95 % CI 5.6–9.3) at RVTH, p value < 0.001. The main modes of transport to the hospital were taxi and ambulance at both sites (Additional file 1: Table S8).

**Table 3:** Out-of-pocket costs before, during and after treatment by study site US$

	Before^e^ mean, median (IQR)	During mean, median (IQR)			After^d^ mean, median (IQR)	Total mean, median (IQR)
		Meals^a^	Visitors gift^b^	Visitors fare^c^		
Basse						
Outpatient pneumonia n = 50	1.7, 0 (0–1.8)	0.1, 0 n/a	n/a	n/a	0.8, 0 (0–1.5)	1.7, 0.02 (0–2.2)
Inpatient pneumonia n = 94	3.3, 1.5 (0–4.2)	5.1, 3.7 (1.7–7.0)	6.5, 4.7 (1.8–10.1)	5.2, 3.3 (1.1–7.3)	2.4, 1.1 (0–2.9)	20.0, 16.8 (9.9–25.0)
Pneumococcal sepsis n = 32	4.9, 3.3 (0–7.6)	9.3, 9.3 (3.4–11.9)	22.3, 7.1 (1.5–10.0)	7.5, 5.3 (0–12.3)	2.3, 1.8 (0–3.9)	44.0, 28.6 (15.2–39.5)
Bacterial meningitis n = 21	3.3, 3.3 (0–4.4)	9.0, 4.9 (0.9–11.6)	6.2, 3.7 (0–8.3)	5.7, 3.7 (0–9.2)	2.5, 1.8 (0–3.7)	30.8, 26.6 (5.3–45.4)
RVTH						
Outpatient pneumonia n = 50	9.6, 4.6 (2.0–11.0)	0.4, 0.02 (0–0.6)	n/a	n/a	1.9, 1.5 (0.7–2.1)	10.0, 4.8 (2.4–12.1)
Inpatient pneumonia n = 81	8.4, 4.1 (2.0–9.9)	6.6, 5.7 (2.9–8.5)	17.4, 14.1 (7.3–21.1)	12.3, 9.1 (4.–15.3)	4.2, 0.8 (0–3.1)	44.2, 40.6 (23.5–58.4)
Pneumococcal sepsis n = 4	25.2, 21.5 (4.4–46.3)	3.5, 2.0 (0–7.0)	8.9, 5.2 (0–17.8)	2.9, 3.1 (0.6–5.3)	24.9, 16.1 (1.6–48.2)	40.6, 38.9 (4.7–66.4)
Bacterial meningitis n = 8	10.9, 8.8 (2.3–14.9)	4.8, 4.4 (2.1–6.1)	26.4, 16.4 (6.8–41.7)	17.9, 11.6 (5.1–24.5)	16.5, 3.5 (0–34.7)	60.0, 57.2 (27.4–90.1)

*IQR* interquartile range

^a^Meals not provided by hospital for patient and care givers

^b^Estimate/actual cost of visitors gifts

^c^To and fro

^d^All costs incurred from up to 1 week after discharge from hospital, assumed transport cost home same as transport cost to the hospital, included in costs after hospital visit

^e^Before includes transport cost to the hospital

In the urban area more than 60 % of health facility visits before hospitalisation occurred at health centres or other regional hospitals. In the rural setting, in addition to health centres, visits to traditional healers (15.3 %) and pharmacies (16.5 %) were common. In the urban area, the average cost for treatment at private clinics was US$19.00. In the rural area, the average cost of treatment from a traditional healer prior to admission was US$8.80 (Additional file 1: Table S9).

Although the hospital provides meals for patients, families also bought food and received gifts from visitors during the hospitalization period. Costs of meals provided by the hospital are incorporated in the bed day costs and are not included in the estimate of costs to the families during hospitalisation. The average out-of-pocket costs incurred before, during and after visits ranged from US$1.70 for OPD pneumonia at Basse to US$60 for inpatient meningitis at RVTH (Table 3). The average after hospital costs highest for pneumococcal sepsis and meningitis at RVTH, US$24.90 and US$16.50 respectively. The average burial costs for ten children at RVTH was US$ 32.80 (Median, IQR = 28.10, 25.70–40.0).

#### Family time lost

The total time lost by caregivers and visitors was estimated for each patient as the time spent at the OPD and/or at the ward. The average time lost by caregivers of hospitalized patients was 1.5 days in Basse and 5 days at RVTH. The majority of the time lost by family members was attributed to time spent by visitors, with an average of 4.5 and 11 h in total visitor time per patient at RVTH and Basse, respectively. The maximum number of visitors/caregivers was 40 at RVTH and 28 at Basse. The estimated loss of income per day ranged from US$22 for paid jobs to US$1 for those who would pay someone to look after their children at home while in hospital (Additional file 1: Table S10). The average estimated loss of income for time spent taking care of the sick child after discharge was US$8.10 [median, IQR 1.18, (0.18–4.15)]. Caregivers lost US$7.00 and visitors about US$1 on average per case.

### Total costs associated with pneumococcal diseases

On average, provider costs for treating outpatient pneumonia, inpatient pneumonia, pneumococcal sepsis and bacterial meningitis were US$8, US$64, US$87 and US$124 per patient respectively. Including out-of-pocket costs, the costs from a societal perspective without considering productivity loss was US$14 and US$96 for pneumonia treated as an outpatient and inpatient respectively, US$130 for pneumococcal sepsis and US$158 for bacterial meningitis. The total societal costs due to outpatient pneumonia, inpatient pneumonia, pneumococcal sepsis and meningitis increased to US$15, US$109, US$144 and US$170 per case respectively when family time loss was taken into account (Table 4).

**Table 4:** Average costs of pneumococcal diseases per patient (US$)

Diagnosis	Total costs by perspective [mean, (median, IQR)]		
	Provider	Societal (includes family OOP)	Societal (family time loss valued)
Outpatient pneumonia	7.6 (6.1, 4.1–8.3)	13.5 (8.6, 5.1–15.2)	15.4 (11.2, 6.4–18.3)
Inpatient pneumonia	64.0 (47.9, 38.2–72.3)	95.5 (79.3,58.9–115.5)	108.6 (87.0, 63.0–129.7)
Pneumococcal sepsis	86.6 (76.5, 50.1–118.8)	130.2 (102.2, 71.8–172.3)	143.7 (122.3, 74.3–193.6)
Bacterial meningitis	123.6 (121.6, (74.7–138.9)	157.6 (139.4, 117.6–198.2)	170.3 (150.1, 117.9–233.2)

IQR inter quartile range, *OOP* out of pocket costs

### Total costs associated with meningitis sequelae

A total of 70 children were identified for follow-up in the study of the economic burden of sequelae following meningitis. Forty-seven (67 %) of these children were traced, eleven had died (23 % CFR) two had travelled out of the country, and two did not consent. In total, twenty-five children, 18 from Basse and 9 from RVTH were followed up.

The mean duration between the onset of meningitis episodes and the interview was 27 months (includes time on admission and maximum 24 months after discharge). The average age of the children was 34 months, (median, IQR 25, 14–38). The average cost to the families for taking care of the children during the acute phase was US$35.6; (US$21.30 in Basse and US$61.00 at RVTH). The average costs due to disability over the first 6 months period after discharge was US$26.0 and the average cost per patient (acute phase and disability) was US$62.5 (Table 5). The disability of the children persists and may last a lifetime therefore projecting the costs the average will be about US$50 by the end of the year and US$2750 over their entire life (median age, 3 years and life expectancy 58 years).

**Table 5:** Average, median, (IQR) out-of-pocket costs to families of children with meningitis (US$)

Costs	Acute^a^	Disability^b^	Total
Basse	21.32, 12.4 (8.4–19.7)	12.0, 0 (0–7.5)	33.3, 15.2 (8.4–37.9)
RVTH	61.0, 33.3 (18.0–45.7)	53.2, 31.2 (12.9–39.3)	114.2, 66.5 (30.9–85.0)
Total	35.6, 18.4 (9.9–37.5)	26.8, 7.2 (0–31.2)	62.5, 30.8 (9.9–66.9)

IQR inter quartile range

^a^Costs before, after and up to 1 week after the acute episode

^b^Costs incurred as a result of disability

Fifteen out of the twenty-seven (55.6 %) children examined had at least one form of neurological deficit (data not shown). On average, a child could not perform any of their usual activities for a period of 10 days (range 1-90) after the onset of the episode of meningitis, and for 28 days (range 0–480 days) children could not perform some activities. Only three households had a family member that was unable to work because of the need to care of the disabled child. The estimated mean loss of earnings per household was US$2.79 over 6 months which translates to an average of US$174 throughout the life of the child. On average, each household spent less than 1 day per month at health facilities providing care due to the child’s disability.

### Sensitivity analysis

A sensitivity analysis was conducted based on varied utilization of diagnostic tests at the two facilities. The first scenario was based on the rates of diagnostic testing at RVTH where only 2 % (2/93) of inpatients had a blood culture. We thus assumed that 2 % of inpatients in Basse had a blood culture. Under this scenario, the total cost for blood culture in Basse declined from US$2250 to US$50 (Table 6). There were no X-rays or blood culture for patients with pneumonia treated at outpatient from both study sites because cases are treated based on clinical diagnosis. Therefore, the costs associated with X-rays and blood culture was zero for outpatient cases. In scenario 2, the average cost per case for diagnostic tests in the MRC laboratory and private laboratories was similar, whereas the RVTH lab costs which are subsidized were lower (Table 6).

**Table 6:** Scenario analysis using different proportion and costs of diagnostic tests at the two facilities

	RVTH N = 93	Basse N = 147
*Scenario 1*		
X-ray		
Number of x-rays	59 (60)	118 (80)
Total cost	18.3	365.8
Proportion who had a diagnostic test		
Assuming 60 % of inpatients at Basse had an x-ray	–	273.4
Assuming 80 % of inpatients at RVTH had an x-ray	231	–
Blood culture		
Number of blood culture	2 (2)	126 (85)
Total cost	335	2252
Cost based on percentage of inpatients at RVTH that had blood culture	–	53
Cost based on percentage of inpatients at Basse that had blood culture	1425	–
*Scenario 2*		
Costs for diagnostic tests from MRC, RVTH or private laboratories		
MRC lab costs		
Outpatient pneumonia	6.5, 5.4 (5.4–8.5)	2.98, 3.1 (1.9–5.0)
Inpatient pneumonia	13.3, 8.5 (5.4–8.5)	28.2, 23.2 (21.1–26.5)
Pneumococcal sepsis	11.5, 6.9 (5.4–17.6)	48.8, 26.6 (23.2–73.0)
Bacterial meningitis	28.4, 11.9 (5.4–56.2)	85.2105.4 (69.9–118.0)
RVTH lab costs		
Outpatient pneumonia	1.28 (0.08, 0.08–3.18	1.63 (3.1, 0.04–3.1)
Inpatient pneumonia	8.74 (3.18, 0.06–3.18)	15.80 (9.57,9.53–9.61)
Pneumococcal sepsis	2.91 (1.63, 0.08–5.75)	35.5 (9.61, 7.41–59.57)
Bacterial meningitis	21.30 (4.10, 0.08–51.9)	75.5 (100.08, 56.47–106.43)
Private lab costs		
Outpatient pneumonia	5.01, 3.68 (3.68–6.78)	2.64, 3.1 (1.84, 3.68)
Inpatient pneumonia	11.95, 6.78 (3.78, 6.78)	21.57, 15.97 (14.13–17.81)
Pneumococcal sepsis	8.45, 5.23 (3.68, 13.21)	41.08, 18.74 (14.71–65.94)
Bacterial meningitis	26.05, 10.18 (3.68–54.60)	79.95, 103.65 (62.87, 111.03)

## Discussion

In this study, we have filled an important evidence gap by estimating the costs of a range of pneumococcal diseases, from a societal perspective, in a typical sub-Saharan Africa setting. We have included the costs to the health care provider, out-of-pocket costs incurred by families, productivity loss and also assessed the economic burden associated with sequelae following bacterial meningitis.

The provider costs associated with the treatment of pneumococcal diseases in the Gambia are substantial, ranging from US$5 for outpatient pneumonia to a peak of US$130 for hospitalized pneumonia patients in urban settings. Out-of-pocket costs to families are also high, with expenditure between $2 for children treated for pneumonia as outpatients and US$60 for children with meningitis.

The average provider costs for one case of outpatient pneumonia was US$8. Outpatient pneumonia is the most common of the disease categories and therefore despite the cost per case being relatively small, the overall cost to the provider is substantial. At RVTH, there are approximately 30 cases of outpatient pneumonia for each case of meningitis (median cost US$85), consequently the economic burden due to outpatient pneumonia was more than five times that of meningitis. In the case of meningitis, the average cost for treating one case was higher than that for inpatient pneumonia nevertheless, given that the number of pneumonia admissions is 15 times that of meningitis admission, the overall economic burden for pneumonia admissions was about ten times greater.

Similar to other African studies, meningitis was the most expensive condition to treat [6, 18]. In Kenya, the cost of meningitis treatment was estimated to be over $160 in a district hospital and over $200 in the central national hospital [6]. Our findings estimated a somewhat lower cost for treatment of meningitis, which is likely due to differences in the available diagnostic tests, costs of resources, and admission policies in Kenya and The Gambia. Treatment costs of pneumonia at OPD derived from this study was also lower than that in Zambia [7]. Pneumonia treatment costs were greater at RVTH whereas treatment costs for sepsis and meningitis were higher in Basse. This may be explained in part by the length of stay, which was longer at RVTH than Basse for inpatients. For OPD pneumonia, the difference was due mainly to the cost of diagnostic tests.

The treatment protocol for pneumonia and meningitis was similar across all surveyed sites; outpatient pneumonia was treated with cotrimoxazole or amoxicillin, while meningitis was treated with benzyl penicillin and chloramphenicol. The availability of X-ray and microbiology tests however varied between locations as did the provision of food for patients. The health facility survey showed however that national treatment guidelines for pneumonia and meningitis are used throughout the country, therefore our estimates for drug costs can be generalized within the country.

Studies in Colombia and India have shown differences in treatment costs at tertiary facilities compared to the secondary and primary level facilities [19, 20]. This may be due to a certain extent to cost of diagnostic tests which are routine in some secondary/tertiary hospitals in other developing countries. Because of the presence of the surveillance study in Basse we did not expect to see a difference between Basse and RVTH. We noted that blood cultures, analysis of cerebrospinal fluid and chest X-rays which are routinely conducted by the pneumococcal disease surveillance system in Basse were not always available at RVTH.

We showed that provider costs represent 50 to 70 % of the societal costs. Out-of-pocket costs ranged from one to ten times of the average daily household expenses (US$5 to US$9). Daily household expenses were higher in Basse where the household size was larger.

The findings of this report have a number of limitations. We enrolled few cases of pneumococcal sepsis and bacterial meningitis. Sepsis cases were particularly low at RVTH reflecting the lack of reliable microbiology services. We identified a total of 36 cases of pneumococcal sepsis and the median costs for diagnostic tests were US$49 and US$12 at Basse and RVTH. Most of the pneumococcal sepsis cases occurred in children admitted with pneumonia, so it was not unexpected that the costs of these two conditions, inpatient pneumonia and sepsis were similar. We included all cases of bacterial meningitis identified during the study period. Of the 29 bacterial meningitis cases, the mean provider treatment costs ranged from US$104 to US$131. In general, antibiotic treatment duration for pneumococcal meningitis is longer than for other bacteria [21]. The same first line antibiotics were used in treatment of meningitis throughout the country as seen from the health facility survey, again giving confidence that the treatment costs can be generalized across the country. Drugs for public health facilities are obtained from one central source.

The economic burden of pneumococcal diseases may be underestimated since we know that not all cases present to health facilities [22]. A health utilization survey has been conducted in URR and data will be used to adjust for children who do not present to health facilities in future analyses of cost effectiveness. Our data may also be extrapolated to estimate treatment costs in adults since the disease burden of pneumococcal diseases is greatest in the very young and the elderly [23], and the antibiotics used for treatment are similar. We expect a greater disease burden if disease in adults is taken into consideration. Cost-effectiveness analyses including adults may need to adjust for length of stay in hospital which may be longer in adults [24].

The study excluded some cost components, such as costs of buildings, equipment, maintenance, and utilities from the main analysis. We did not collect burial costs at Basse however, if we extrapolate the average burial cost for children at RVTH to Basse, the family out-of-pocket cost increases by another US$650.

Our difficulty in tracing children for follow-up after pneumococcal meningitis is likely to reflect the high mortality associated with this condition. Even though we confirmed 11 deaths, it is possible that more of the children not traced (23/70) had died. For those children that were followed up, the estimated monthly costs of providing care for the child was around US$3. For one in ten families, one family member was not able to work due to the need to provide special levels of care for the child.

Clearly, pneumococcal meningitis continues to be associated with substantial economic and social burdens for families, even when children survive the acute illness. Meningitis is such a dramatic event that mothers would remember details of their child’s illness but we cannot rule out recall bias. Mothers were to give costs at the time of the illness but it is possible that they stated current costs rather than actual costs incurred at the time of the incidence resulting in overestimation of costs due to inflation over time.

Our findings demonstrate that pneumococcal disease in The Gambia is associated with substantial economic costs of more than a US$100 for admitted cases. The health system meets 50 to 80 % of these costs. Out-of-pocket costs were one to ten times the average daily household expenditure. Compared to Kenya and Pakistan, fewer costs were passed on to caregivers by the health system [6, 18]. These are timely data to support investments in prevention and treatment programmes, and will be used for cost-effectiveness analyses of the introduction of pneumococcal conjugate vaccine.

The ultimate aim of economic analysis is to improve and support decisions around allocation of health resources. Understanding the costs associated with treating pneumococcal disease highlights the magnitude of the costs that can be averted when these diseases are prevented. The economic burden numbers presented in this analysis are, in aggregate, smaller than are reported in similar analyses in other larger countries. However, the economic burden of childhood pneumococcal disease is significant both for households and for the country as a whole. From the household perspective, out-of-pocket costs ranged from one to ten times daily household expenditures. These costs can be dramatic for the economically disadvantaged. Likewise, the economic burden of one case of inpatient pneumonia (US$109) is much larger than The Gambia’s health expenditure per capita of US$29 [25]. These numbers only provide partial insight into the economic burden of pneumococcal disease in The Gambia. Nonetheless, they demonstrate the challenges faced by households and the country as a whole in dealing with one of the leading causes of childhood mortality.

## Conclusions

The economic burden of pneumococcal diseases in The Gambia is substantial. Apart from the significant costs to the health care providers, families of children with pneumococcal diseases incur considerable expenses during the course of treating the sick child. The introduction of pneumococcal conjugate vaccine has the potential to significantly reduce this economic burden.

## Additional file

10.1186/s12962-016-0053-4 Addtional Supporting Tables.

## Abbreviations

BCG: Bacillus Calmette–Guérin
CHOICE: choosing -effective interventions that are cost
EPI: expanded programme on immunisation
IQR: inter quartile range
MRC: Medical Research Council
OPD: out patient department
PCV: pneumococcal conjugate vaccine
RVTH: Royal Victoria Teaching Hospital
URR: upper river region
