# Supplementary material for: The economic burden of childhood pneumococcal diseases in The Gambia
Source: Cost Eff Resour Alloc. 2016 Feb 17;14:4. doi: 10.1186/s12962-016-0053-4 (PMC4758012; doi:10.1186/s12962-016-0053-4)
Supplement: Supplementary file 1 — 10.1186/s12962-016-0053-4 Addtional Supporting Tables. [file 12962_2016_53_MOESM1_ESM.docx]

**Supplementary tables**

**Supplementary Table 1**: Estimated costs of medications provided in health facilities

| Drug name | Preparation | Unit | Amount | Cost ($US) |
| --- | --- | --- | --- | --- |
|  |  |  |  |  |
| Antibiotics |  |  |  |  |
|  |  |  |  |  |
| Ampicillin | injection | 2ml vial | 500mg | 0.3 |
| Ampicillin | syrup | 100ml bottle | 125mg/5ml | 1.2 |
| Chloramphenicol | injection | 2ml vial | 1g | 0.5 |
| Chloramphenicol | syrup | 100ml bottle | 125mg/5ml | 0.94 |
| Metronidazole | injection | 10ml bottle | 5mg/ml | 1.99 |
| Metronidazole | syrup | 100ml bottle | 125mg/5ml | 0.61 |
| Cotrimoxazole | syrup | 100ml bottle | 40/200mg/5ml | 0.83 |
| Cotrimoxazole | tablet | 1000/tin | 120mg/tab | 14.2 |
| Amoxycillin | syrup | 100ml bottle | 125mg/5ml | 0.64 |
| Gentamicin | injection | 2ml vial | 80mg | 0.13 |
| Penicillin | injection | 2ml vial | 1,000,000IU  600mg | 0.26 |
| Ampi-cloxacillin | syrup | 100ml bottle | 125gm/5ml | 0.34 |
| Ceftriaxone | injection | 2ml vial | 250mg | 2.5 |
| Ciprofloxacin | injection | 100ml bottle | 2mg/ml | 3.93 |
| Ciprofloxacin* | tablet | 100/packet | 250mg | 2.71 |
| Erythromycin* | syrup | 100ml bottle | 125mg/5ml | 1.47 |
| Penicillin V* | syrup | 100ml bottle | 125mg/5ml | 1.08 |
| Cloxacillin* | injection | 2ml vial | 500mg | 13.71 |
|  |  |  |  |  |
| Other drugs |  |  |  |  |
|  |  |  |  |  |
| Paracetamol | syrup | 500ml bottle | 120mg/5ml | 0.44 |
| Paracetamol | tablet | 1000/tin | 100mg/tab | 5.0 |
| Multivitamin | syrup | 100ml bottle |  | 1.0 |
| Multivitamin | tablet | 1000/tin |  | 6.66 |
| Salbutamol | syrup | 100ml bottle | 2mg/5ml | 2.25 |
| Salbutamol | tablet | 1000/tin | 4mg/tab | 9.8 |
| Quinine | injection | 2ml vial | 600mg | 1.3 |
| Frusemide | injection | vial |  | 0.12 |
| Aminophylline | injection | vial |  | 0.3 |
| Piriton | syrup | bottle |  | 1.0 |
| Oral rehydr. soltn | sachet |  |  | 0.5 |
| Hb12 | syrup | bottle |  | 0.79 |
| Folic acid | tablet | 1000/tin |  | 6.67 |
|  |  |  |  |  |
| IV fluids |  |  |  |  |
|  |  |  |  |  |
| Ringer’s lactate | liquid | 500ml |  | 0.55 |
| Normal saline | liquid | 500ml |  | 0.5 |
| 5% glucose | liquid | 500ml |  | 0.55 |
|  |  |  |  |  |
| Drug delivery |  |  |  |  |
|  |  |  |  |  |
| Needles |  | 100 |  | 8 |
| Syringes |  | 100 |  | 8 |
| 70% alcohol |  | 200ml bottle |  | 2.33 |

Cost data from National central stores

*cost estimated from Durbin, UK

**Supplementary Table 2:** Costs of diagnostic tests at MRC, RVTH and two private laboratories

| **Diagnostic test** | **Laboratory name** | | | |
| --- | --- | --- | --- | --- |
|  | MRC ($US) | RVTH ($US) | ‘Medlab’ ($US) | ‘Mamadi’s’ ($US) |
| Full blood count | 6.84 | 1.84 | 6.43 | 6.80 |
| Haemoglobin | 3.44 | 0.04 | 1.84 | 1.84 |
| Blood culture | 18.02 | 6.43 | n/a | 11.03 |
| Blood film | 1.92 | 0.04 | 0.04 | 1.84 |
| CSF examination | 46.88 | n/a | n/a | n/a |
| Electrolytes | 4.10 |  |  |  |
| X-ray | 1.92 | 3.10 |  |  |

**Supplementary Table3:** Resource use at RVTH and Basse health centre

| **Resource** | **RVTH** | **Basse** |
| --- | --- | --- |
| Number of beds ^a^ | 390 | 57 |
| Number of paediatric beds ^b^ | 41 | 16 |
|  |  |  |
| Bed occupancy ^c^ Paediatric | 0.88 | 0.95 |
| Adults | 0.5 | 0.5 |
| Personnel costs |  |  |
| One bed day | 2.78 | 4.10 |
| One patient day | 3.17 | 4.33 |
| Meals cost per day | 0.92 | 0.91 |
|  |  |  |
| *Paediatric patient day cost* | *4.37* | *5.29* |

^a^ Number of beds at RVTH is an estimate

^b^ Excludes neonatal and surgical beds, costs US$ (2011)

^c^ Bed occupancy was calculated as the total number of admissions multiplied by the average duration of admission in days, divided by the number of beds multiplied by 365

**Supplementary Table 5**: Educational background of caregivers

|  | **Primary**  **N (%)** | **Secondary**  **N (%)** | **Tertiary**  **N (%)** | **Arabic**  **N (%)** | **None**  **N (%)** | **Total**  **N (%)** |
| --- | --- | --- | --- | --- | --- | --- |
| **Basse** | 17(8.6) | 10(5.1) | 2(1.0) | 138(70.1) | 28 (14.2) | 195/197 |
| **RVTH** | 19(13.3) | 23(16.1) | 13(9.1) | 73(51.1) | 12 (8.4) | 140/143 |
| **Total** | 36(10.6) | 33(9.7) | 15(4.4) | 211(62.1) | 40 (11.8) | 335/340 |

*5 missing

**Supplementary Table 6**: Normal daily household expenses, 2011-12

|  | **Food**  **Mean, median (IQR)** | **Rent**  **Mean, median (IQR)** | **Electricity & Water**  **Mean, median (IQR)** | **Total expenses**  **Mean, median (IQR)** |
| --- | --- | --- | --- | --- |
| **Basse**  N=197 | 9.11, 7.35 (3.68-11.03) | 0.06, 0 (0-0) | 0.04,0 (0-00 | 9..05, 7.35 (3.68-11.03) |
|  |  |  |  |  |
| **RVTH**  N=143 | 5.23,3.68 (2.76-5.51) | 0.49, 0 (0-0.61) | 0.32, 0 (0-0.25) | 5.38, 3.68 (2.76-5.88) |

**Supplementary Table 7**: Percentage of Households able to stock commodities in the previous month before the survey

| Item | Always | Sometimes | Never |
| --- | --- | --- | --- |
|  | N (%) | N (%) | N (%) |
| **Basse** |  |  |  |
| Rice | 122(65.0) | 45(24.0) | 20(11.0) |
| Oil | 53(28.3) | 61(32.6) | 73(39.1) |
| Others | 30(16.0) | 55(29.4) | 102(54.6) |
| **RVTH** |  |  |  |
| Rice | 51(36.7) | 52(37.4) | 36(25.9) |
| Oil | 15(10.9) | 27(19.6) | 96(69.6) |
| Others | 7(5.1) | 25(18.3) | 105(76.6) |

**Supplementary Table 8**: Mean cost incurred before visiting the health facility US$

|  | **Mean Costs** | | | | |
| --- | --- | --- | --- | --- | --- |
|  | **Transport*** | **Consultation** | **Drugs** | **Diagnostics** | **Total,**  **median**  **(IQR )** |
| **Basse** |  |  |  |  |  |
| Outpatient Pneumonia n=50 | 0.28 | 0.01 | 0.70 | 0 | 1.08,0  (0-0.74) |
| Inpatient Pneumonia n=94 | 0.42 | 0.02 | 1.24 | 0.01 | 1.74, 0  (0-1.84) |
| Penumococcal Sepsis n=32 | 0.71 | 0 | 1.39 | 0 | 2.09, 0  (0-1.6) |
| Bacterial Meningitis n=21 | 0.80 | 0.02 | 0.50 | 0 | 1.32,0  (0-2.6) |
| **RVTH** |  |  |  |  |  |
| Outpatient Pneumonia n=50 | 2.45 | 0.47 | 3.81 | 0.15 | 7.96, 2.6  (0.6-9.2) |
| Inpatient Pneumonia n=81 | 2.29 | 0.47 | 2.95 | 0.07 | 6.57, 2.3  (0.7-7.3) |
| Pneumococcal Sepsis n=4 | 5.94 | 2.39 | 5.47 | 0 | 16.46,14.5 (2.0-30.9) |
| Bacterial Meningitis n=8 | 2.03 | 0.57 | 5.70 | 0 | 9.65,6.4  (1.9-12.9) |

*All transport prior to this hospital visit does not include cost of transport to the hospital

**Supplementary Table 9**: Mean out of pocket costs incurred at other health facilities visited before visit to study site US$

| **Facility** | **Basse** |  | **RVTH** |  |
| --- | --- | --- | --- | --- |
|  | N (%) | Total mean cost | N (%) | Total mean cost |
| Hospital | 1(0.6) | 0.18 | 133(38.2) | 10.6 |
| Health centre | 89(52.3) | 1.90 | 116(33.3) | 11.7 |
| Private | 7(4.1) | 5.75 | 29(8.3) | 19.0 |
| Pharmacy | 28(16.5) | 5.00 | 49(13.8) | 14.5 |
| Traditional healer | 26(15.3) | 8.82 | 14(4.0) | 11.3 |
| Shop | 7(4.1) | 1.05 | 1(0.3) | 0.59 |
| Other | 12(7.1) | 2.67 | 7(2.1) | 25.2 |

**Supplementary Table 10**: Estimated loss of income due to time loss for different types of job US$ per day

| Activity | Basse  Mean, median IQR | RVTH  Mean, median IQR |
| --- | --- | --- |
| Housework^a^ | 3.7,0  (0-3.7) | 10.1,3.7  (0-11.0) |
|  |  |  |
| Income Paid job | 10.4, 5.5  (0.5-13.8) | 22.4, 11.0  (0.2-34.1) |
| Looking after children^b^ | 1.0, 0  (0-0) | 4.1, 1.1  (0-5.5) |

^a^ estimate of how much to pay for housework to be to be done per day

^b^ estimate of how much to pay to look after children per day
